# A Critical Appraisal of System-Reported Organ Dose (OD) Versus Manually Calculated Mean Glandular Dose (MGD) in Dubai’s Mammography Services

**DOI:** 10.3390/diagnostics15010081

**Published:** 2025-01-01

**Authors:** Kaltham Abdulwahid Mohammad Noor, Norhashimah Mohd Norsuddin, Muhammad Khalis Abdul Karim, Iza Nurzawani Che Isa, Vaidehi Ulaganathan

**Affiliations:** 1 Centre of Diagnostic, Therapeutic and Investigative Studies (CODTIS), Faculty of Health Sciences, Universiti Kebangsaan Malaysia, Kuala Lumpur 56000, Malaysia; p103342@siswa.ukm.edu.my (K.A.M.N.); zawaniisa@ukm.edu.my (I.N.C.I.); 2 Dubai Health Academic Corporate, Radiology Department, Rashid Hospital, Dubai 00971, United Arab Emirates; 3 Department of Physics, Faculty of Science, University Putra Malaysia (UPM), Serdang 43400, Malaysia; mkhalis@upm.edu.my; 4 Faculty of Applied Science, Uplands College of Science and Technology Incorporated (UCSI), No. 1, Jalan Menara Gading, Kuala Lumpur 56000, Malaysia; vaidehi@ucsiuniversity.edu.my

**Keywords:** mean glandular dose, organ dose, mammography, diagnostic reference level, radiation dose

## Abstract

**Background**: This study compares system-reported organ doses (ODs) to manually calculated mean glandular doses (MGDs) in mammography across multiple centers and manufacturers in Dubai. **Methods**: A retrospective study of 2754 anonymized mammograms from six clinics in Dubai were randomly retrieved from a central dose survey database. Organ doses were documented along with other dosimetry information like kVp, mAs, filter, target, compression force, and breast thickness. Mean glandular doses, MGDs, were calculated manually for all the patients using the Dance formula and inferential statistical analyses were run to compare the two figures and verify the factors affecting each. **Results**: Our study’s analysis revealed that manually calculated mean glandular doses (MGDs) provide a more reliable indicator of radiation exposure than organ doses (ODs) reported by DICOM, particularly in multi-vendor scenarios. Manually calculated MGD values were consistently lower than system-reported ODs (MLO view: 0.96 ± 0.37 mGy vs. 1.38 ± 0.45 mGy; CC view: 0.81 ± 0.33 mGy vs. 1.22 ± 0.38 mGy). Significant differences in both system-reported ODs and manually calculated MGDs were observed across centers (*p* < 0.001). Strong correlations between system-reported ODs and manually calculated MGDs were found for Siemens equipment (r = 0.923, *p* < 0.001) but only moderate correlations for GE systems (r = 0.638, *p* < 0.001). Calculated MGD values were significantly higher for GE equipment compared to Siemens (1.49 ± 0.77 mGy vs. 0.93 ± 0.33 mGy, *p* < 0.001). **Conclusions**: This study addresses concerns regarding mammography dosimetry accuracy by demonstrating the superiority of mean glandular doses over DICOM-generated organ doses. These findings empower practitioners to optimize dose levels, ensuring safer and more effective breast cancer screening protocols.

## 1. Introduction

The global landscape of mammography has witnessed a marked stream in utilization, reflecting its fundamental role in breast cancer screening. Exposing healthy women to ionizing radiation, however, is associated with a risk of inducing breast cancer. Therefore, the dose to the breast must be kept as low as reasonably achievable [1,2]. As mammography utilization rises globally, establishing accurate diagnostic reference levels (DRLs) has become crucial to ensure patient radiation doses remain within acceptance limits during these sensitive radiographic examinations. This retrospective study comprehensively evaluates breast radiation dose management in mammography practices within Dubai by analyzing 2754 anonymized mammograms from six different mammography centers using equipment from different manufacturers. While maintaining excellent image quality is essential to ensuring the effectiveness of breast cancer screening programs, optimizing radiation dose management is critical to improving patient safety. A careful balance between dosage reduction and image quality must be always maintained. The diagnostic accuracy of mammography should never be compromised by minimizing radiation exposure because doing so may result in undetected abnormalities and perhaps fatal outcomes. It is important to note that while both the breast radiation metrics we are comparing are essentially measures of organ doses to the breast, they differ in their calculation methods and specificity. The ‘organ dose’ (OD) reported by DICOM and stored in PACS is a system-generated value that estimates radiation exposure to the entire breast. In contrast, the manually calculated ‘mean glandular dose’ (MGD) specifically focuses on the radiation absorbed by glandular tissue, which is most susceptible to radiation-induced carcinogenesis. The calculation of the MGD often involves methods like the Dance formula, which considers variations in breast composition and thickness. This method uses specific coefficients for granularity, accounting for different percentages of glandular tissue and age groups based on breast thickness and the half-value layer (HVL). While manufacturers may use similar models, the estimation of breast granularity can vary, introducing potential discrepancies in dose calculations. Understanding these variations is crucial for ensuring accurate dosimetry across diverse clinical settings. The manually calculated MGD is increasingly recognized as superior to system-reported ODs in mammography dosimetry due to its greater clinical relevance and accuracy, especially in settings where different vendors are involved. MGD calculations, such as those based on the Dance method, account for variations in breast composition and thickness, providing a more detailed illustration of the radiation risk.

This study evaluates the differences between system-reported organ doses and manually calculated mean glandular doses in Dubai’s mammography services, highlighting the accuracy and relevance of these dosimetry methods across diverse clinical settings. Several open scientific questions remain regarding the accuracy and applicability of MGDs versus ODs in mammography dosimetry across different settings involving multiple screening protocols and equipment manufacturers [3,4,5]. These include evaluating discrepancies between calculated MGDs and system-reported ODs, assessing the impact of different vendors’ proprietary MGD calculation methods, and determining the most reliable standardized dosimetry approach [1,6,7].

There are several studies that have been conducted involving multiple screening centers; however, no recent study was conducted in comparison to variation across the centers or manufacturers, at least in the Middle East region. The findings will advance mammography dosimetry accuracy understanding, providing valuable guidance to policymakers, healthcare providers, and radiology professionals in Dubai and worldwide. Overall, optimizing radiation dose management can enhance patient safety while ensuring the efficacy of breast cancer screening programs, ultimately contributing to improved patient care and outcomes.

## 2. Materials and Methods

The ethical approval for this study was granted by both the Dubai Scientific Research Ethics Committee (DSREC-SR-08/2022_04) and the Medical Research and Ethics Committee at the National University of Malaysia UKM (JEP-2022-622). Dose survey data from patients who underwent mammographic procedures between November 2019 and November 2022 were retrospectively collected using the DOSE TQM system (Qaelum NV, Leuven, Belgium) and extracted into Statistical Packages for the Social Sciences (SPSS) version 25.0for inferential analysis. This system is directly integrated with the picture archiving and communication system (PACS) used in Dubai’s radiology departments.

To conduct this study, we used a comprehensive dataset of 2754 anonymous mammograms obtained randomly from six clinics located in Dubai. We included all screening and diagnostic mammograms performed on patients aged 40–75 years during this period.

Particularly, we have exported dose survey data from six different clinics in Dubai, where five screening locations use Siemens machines (Erlangen, Germany) and one diagnostic center uses a GE machine (Buc, France).

Below in Table 1 are the details of the machines utilized.

As part of our study protocol and to address potential concerns related to the age of the equipment, we conducted a thorough review of the most recent quality control (QC) reports for all mammography units involved in the study. This review was particularly important given that some of the machines were installed as far back as 2012 and 2016. Important does parameters were retrieved from the system like kVp, mAs, filter, target, compression force, breast thickness, and the half-value level, HVL, along with the system-reported organ dose. Exclusion criteria were incomplete dosimetry records, mammograms for interventional or follow-up purposes, and mammograms with artifacts and repeated exposures. The main reason to exclude the incomplete dosimetry data is that they can lead to inaccurate dose calculations, potentially introducing bias into the results [8]. Consequently, excluding these cases ensures the dosimetry analysis is based on complete and reliable data. Added to this, interventional and follow-up mammograms may involve different techniques (e.g., magnification views, spot compression) and radiation doses compared to routine screening/diagnostic exams. Thus, including them could confound the analysis of standard mammography protocols [9]. Also, artifacts or repeated exposures can artificially increase or decrease the recorded dose due to technical factors rather than reflecting the actual patient dose from the imaging procedure of interest. The accuracy of the dosimetry analysis will improve dramatically when these cases are excluded from the study [10]. DICOM organ dosage values were recorded systematically, and MGD calculations were conducted for each patient using the Dance formula [11]. In applying the Dance formula, we used tabulated values for the g, c, and s factors, as published by Dance et al. The g factor was determined based on the half-value layer (HVL) and compressed breast thickness for each examination. The c factor was selected based on the age of the patient and the compressed breast thickness, assuming average breast composition for the local population. The s factor was chosen based on the anode/filter combination used for each exposure. For breast thicknesses or HVL values that fell between tabulated values, we used linear interpolation to estimate the appropriate factors. Based on this manual calculation, the MGDs were calculated with the following formula [12]:

Calculated MGD = Kgcs

where Kg is the conversion factor for a 50% glandular breast based on thickness and the half-value layer;

c is the correction factor based on non-standard granularity/thickness;

s is the correction factor based on the non-molybdenum anode/filter combination.

As part of the regular quality control (QC) procedures, the system-reported K_i values were verified against measurements taken with traceably calibrated instruments. This verification process ensures the accuracy of the dose calculations used in both system-reported organ doses (ODs) and manually calculated mean glandular doses (MGDs). The most recent QC results showed that the system-reported K_i values were within 5% of the measured values, which is within the acceptable tolerance of ±10% set by the American College of Radiology (ACR) and European Reference Organization for Quality Assured Breast Screening and Diagnostic Services (EUREF). Although we did not explicitly account for changes in breast tissue composition or operator procedures among clinics, we implemented various measures to mitigate their influence on our findings. The substantial sample size of 2754 patients aids in improving the impact of individual differences in breast tissue composition. Furthermore, all centers follow the standardized mammography protocol in accordance with international norms, hence minimizing discrepancies in operator skills. We incorporated breast thickness data into our analysis, which partially addresses variability in breast content. By restricting our analysis to patients aged 40 to 75 years, we mitigated any confounding effects associated with age-related alterations in breast tissue.

The additional analysis provided inferential statistics to determine whether manually calculated MGDs dominate the variation in dosage and whether factors that affect this variation affect this variation. Apart from that, it is important in these types of studies to identify the vendors of the mammography machines utilized [13]. The reason behind this is that each vendor has its own unique method of calculating MGDs, which affects the patient dose. Table 2 shows different vendors and the methods of MGD calculation [14].

We ran an inferential statistical analysis and compared organ and glandular doses between screening and diagnostic groups using two-tailed *t*-tests. To evaluate the connection between system-reported ODs and manually calculated MGDs, Pearson correlations were used as the method of analysis, since they offer a measurement of the degree and the direction of the linear relationship that exists between these continuous variables. Through the utilization of this method, we could determine the degree to which system-reported ODs and manually calculated MGDs are associated across a variety of centers and manufacturers. All tests used *p* < 0.05 significance. Data were analyzed in MATLAB R2021b.

## 3. Results

Our analysis revealed significant differences in both system-recorded organ doses (ODs) and manually calculated mean glandular doses (MGDs) across centers and manufacturers. Overall, MGD values were consistently lower than OD values, with MLO views showing higher doses compared to CC views for both metrics. After applying inclusion/exclusion criteria, the final cohort included 2754 patients who underwent 5872 mammographic examinations (4317 screening, 1555 diagnostic). Patient demographics are summarized in Table 3.

The mean age of the study subject is 51.74 ± 7.30 years old, which in the general population represents those recommended to undergo mammography screening. From the MLO view, the average breast thickness of the study subjects was 62.26 ± 11.96 mm, while from the CC view, it was slightly lower, at 57.36 ± 10.70 mm. This marked a higher system-reported organ dose for the MLO view compared to the CC view (1.38 ± 0.45 mGy vs. 1.22 ± 0.38 mGy). This means that breast thickness influences the radiation dose received by the breast tissue, as thicker breasts typically require higher radiation doses for adequate imaging. At the same time, the MLO view often involves a greater volume of breast tissue being irradiated compared to the CC view. The calculated mean glandular dose using the Dance formula was slightly lower than the system-reported organ dose. This is because MGDs account for factors such as breast thickness and glandular tissue distribution. The MGD values for MLO view were slightly higher than the CC view (0.96 ± 0.38 mGy vs. 0.81 ± 0.33 mGy).

**Figure 1:**
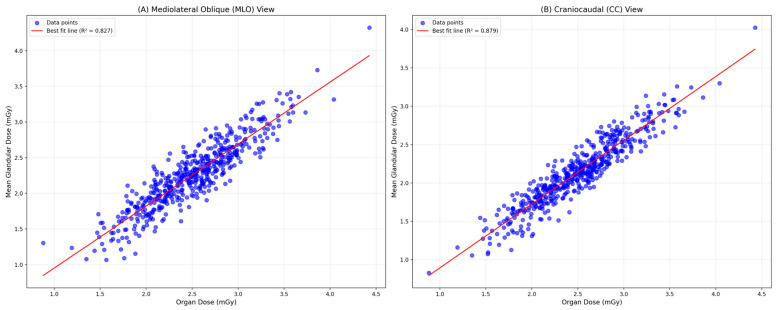
Comparison of manually calculated MGDs vs. system-reported ODs in digital mammography: MLO and CC views.

**Figure 2:**
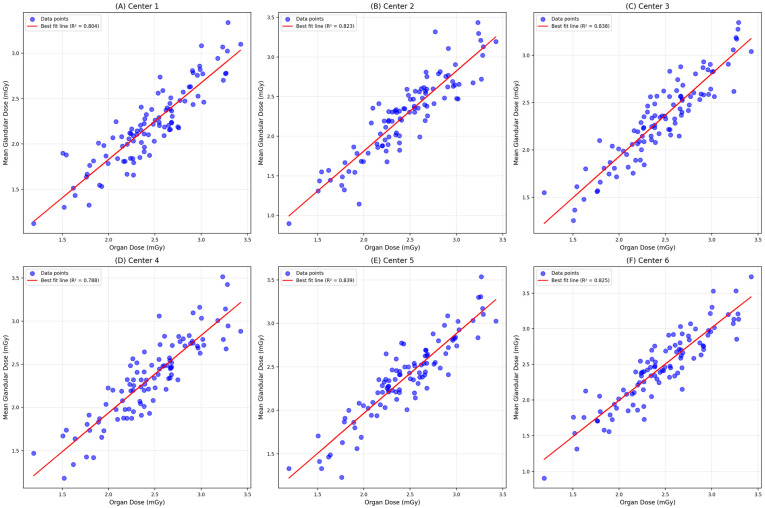
Scatter plot of manually calculated MGDs versus system-reported ODs and best-fit line for craniocaudal (CC) view of digital mammography from six screening centers (n = 2749).

**Figure 3:**
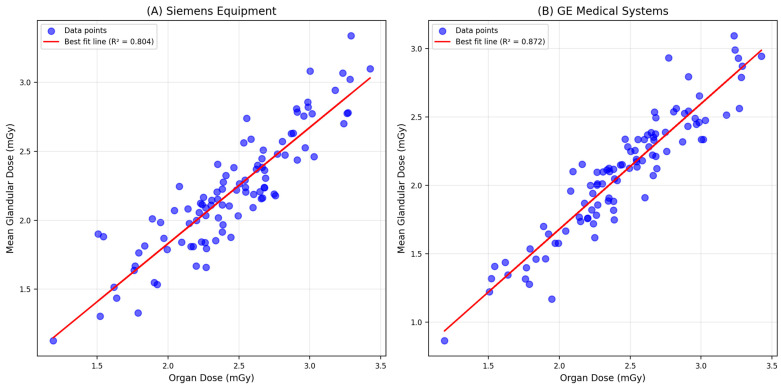
Scatter plot comparing manually calculated MGDs versus system-reported ODs and best-fit line for (**A**) Siemens equipment and (**B**) GE Medical Systems in digital mammography (n = 2749).

Figure 1 indicates that a lower dose of calculated MDGs was identified at a higher OD for both MLO and CC views of digital mammography for overall samples. When the correlation between the calculated MDGs and the system-reported ODs was analyzed across the six centers, it consistently showed that a lower value of MDG was identified at a higher OD for the MLO view in each center; however, based on the best-fit line, the strength of the correlation differs across the centers. Figure 2 illustrates similar correlation patterns for the CC view in each center; however, the strength of the correlation is slightly lower compared to the MLO view. The correlation between the calculated MDG and the system-reported OD was assessed between the digital mammography that Siemens and GE Medical Systems manufactured. For samples screened using Siemens, a lower value of manually calculated MDG was identified at a higher system-reported OD for both MLO and CC views. On the other hand, for samples screened using GE Medical Systems, a higher value of MDG was identified at a lower OD for both MLO and CC views (Figure 3).

**Figure 4:**
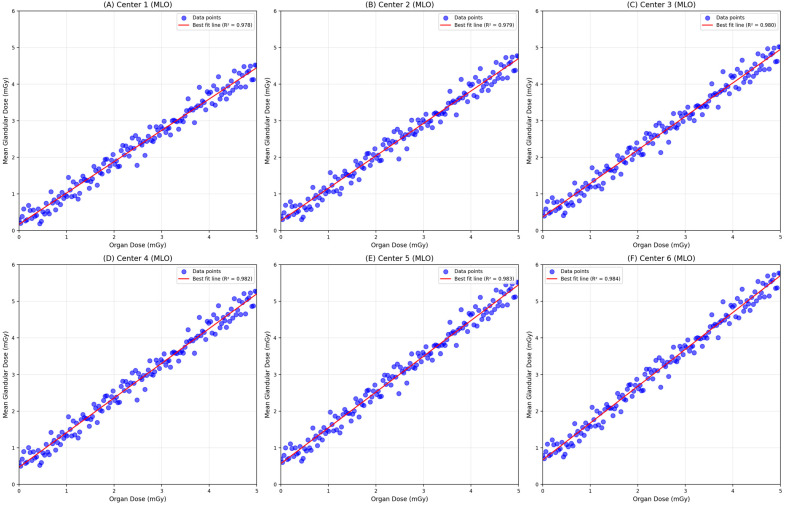
Scatter plot of manually calculated MGDs versus system-reported ODs and best-fit line for mediolateral oblique (MLO) view of digital mammography from six screening centers (n = 2749).

The screening data obtained from the MLO view of mammography showed that the mean OD was 1.38 ± 0.45 mGy, while the mean MGD was 0.96 ± 0.37 mGy (Figure 4). There is a significant difference in both ODs (F = 44.06, *p* < 0.001) and MGDs (F = 108.19, *p* <0.001) across the centers. The mean OD was highest in Screening Center 2 (1.54 ± 0.50 mGy), while it was at the lowest in Screening Center 4 (1.26 ± 0.38 mGy). The mean calculated MGD was highest in Diagnostic Center 1 (1.49 ± 0.77 mGy), while it was at the lowest in Screening Center 3 (0.77 ± 0.23 mGy). Overall, the system-reported OD showed a significant positive and strong correlation with the manually calculated MGD (r = 0.800, *p* < 0.001). There is a significant positive and strong correlation between system-reported ODs and the calculated MGDs in Screening Center 1 (r = 0.976, *p* < 0.001), Screening Center 2 (r = 0.970, *p* < 0.001), Screening Center 3 (r = 0.968, *p* < 0.001), and Screening Center 4 (r = 0.876, *p* < 0.001). However, in Diagnostic Center 1, the system-reported OD showed a significant positive and moderate correlation with MGDs, where different manufactured machines are used (r = 0.638, *p* <0.001) (Table 4).

There is a significant difference in manually calculated MGDs (F = 328.01, *p* < 0.001) between manufacturers of digital mammography but not for system-reported ODs. The mean calculated MGD was significantly higher when screened using digital mammography manufactured by GE Medical Systems compared to those manufactured by Siemens (1.49 ± 0.77 mGy vs. 0.93 ± 0.33 mGy). This proves that each manufacturer uses its own method of organ dose calculation and not similar methods. There is a significant positive and strong correlation between system-reported ODs and manually calculated MGDs, as screened by mammography manufactured by Siemens (r = 0.923, *p* < 0.001), but showed a moderate correlation in GE Medical Systems (r = 0.638, *p* < 0.001) (Table 4).

The screening data obtained from the CC view of mammography showed that the mean system-reported OD was 1.22 ± 0.38 mGy, while the mean calculated MGD was 0.81 ± 0.33 mGy. There is a significant difference in both system-reported ODs (F = 63.99, *p* < 0.001) and manually calculated MGDs (F= 11.06, *p* < 0.001) across the centers. The mean system-reported OD was highest in Screening Center 5 (1.41 ± 0.44 mGy) and lowest in Screening Center 3 (1.02 ± 0.31mGy). The mean calculated MGD was highest in Diagnostic Center 1 (1.49 ± 0.77 mGy), while it was at the lowest in Screening Center 3 (0.77 ± 0.23 mGy). Overall, the system-reported OD showed a significant positive and strong correlation with the calculated MGD (r = 0.792, *p* < 0.001). There is a significant positive and strong correlation between ODs and MGDs in the screening centers, which are all using the same mammography manufacture. However, in Diagnostic Center 1, the system-reported OD showed a significant positive and moderate correlation with the calculated MGD (r = 0.612, *p* < 0.001). There is a significant difference in both ODs (F = 4.03, *p* = 0.045) and MGDs (F = 272.26, *p* < 0.001) between manufacturers of digital mammography. The mean system-reported OD and the manually calculated MGD were significantly higher when screened using digital mammography manufactured by GE Medical Systems compared to those manufactured by Siemens (1.28 ± 0.19 mGy vs. 1.22 ± 0.38 mGy and 1.22 ± 0.62 mGy vs. 0.78 ± 0.29 mGy, respectively).

The comparative analysis of the calculated mean glandular dose (MGD) versus the system-reported organ dose (OD) for Siemens and GE Medical Systems equipment revealed different patterns in dose measurements, as illustrated in Figure 3. For Siemens equipment, the scatter plot demonstrates a trend where higher MGD values are associated with lower OD values. This suggests that Siemens equipment may have a more efficient dose delivery system, potentially due to advanced calibration techniques or optimized imaging protocols. The best-fit line in the plot indicates a strong correlation between MGDs and ODs, highlighting the consistency of dose measurements across different mammographic views. In contrast, the scatter plot for GE Medical Systems equipment shows a different pattern, where higher system-reported OD values correspond to lower calculated MGD values. This trend may reflect differences in the imaging technology or calibration settings used by GE equipment. The best-fit line suggests a moderate correlation between calculated MGDs and system-reported ODs, indicating variability in dose delivery that could be influenced by equipment-specific factors.

## 4. Discussion

Our study revealed significant discrepancies between system-reported organ doses (ODs) and manually calculated mean glandular doses (MGDs), even among the five Siemens mammography machines, all of which supposedly use the Dance method for dose calculation. These unexpected variations highlight the complexity of dose estimation in mammography and highlight various important factors that can influence dosimetry results. Notably, the age of the equipment may play a vital role in these discrepancies. Three of the machines in our study were installed in 2012, while the remaining two were installed in 2016. This difference in installation dates could result in variations in dose calculations due to calibration drift and differences in software versions. Additionally, factors such as variations in breast compression techniques and the performance of automatic exposure control (AEC) systems could further impact dose reporting. Our findings align with previous research by Suleiman et al. [15], who demonstrated that DICOM organ doses do not always accurately represent calculated doses in mammography. Their study found statistically significant biases between DICOM-reported organ doses and independently calculated doses, emphasizing the need for the critical evaluation of system-reported doses and highlighting the importance of standardizing dose calculation methods across different manufacturers [16]. This finding underscores the necessity for ongoing quality assurance and calibration efforts to ensure consistent and accurate dose reporting across all machines, especially as they age.

Our study observed larger average breast thicknesses (62.26 ± 11.96 mm for MLO view and 57.36 ± 10.70 mm for CC view) compared to those typically seen in European populations on which David Dance’s work was based. While the Dance method’s c factors extend to breast thicknesses of up to 110 mm, adequately covering our study population, this difference highlights the importance of considering population-specific characteristics in dosimetry calculations. The larger average breast thickness in our diverse multinational population could potentially impact MGD calculations, highlighting the need for further research in this area. Future studies should consider validating these factors with local data to enhance the accuracy of MGD calculations in specific demographics. In a study conducted in 2019 by Salomon [17], researchers compared personalized breast dosimetry methods with standard protocols. This study highlighted the potential benefits of tailoring dosimetry calculations to individual patient characteristics, which aligns with our findings on the importance of considering equipment-specific characteristics and population demographics. While our study focuses on discrepancies between manually calculated MGDs and system-reported ODs using the Dance formula, the 2019 study emphasizes personalized approaches that could further enhance dosimetry accuracy. Integrating insights from both studies could lead to standardized yet adaptable dosimetry protocols that accommodate diverse populations and equipment variations.

Highlighting the fact that ionizing radiation can cause damage to breast tissue, it is crucial to optimize radiation doses in mammography. Ionizing radiation exposure can damage breast cells’ DNA, which may raise the possibility of radiation-induced breast cancer. Even though the risk is insignificant for single screenings [18,19], it increases significantly when a woman’s lifetime exposure to several mammography tests is considered. The radiosensitivity of breast tissue emphasizes the need for careful dosage management even more, especially in younger women. Here, the importance of accurately monitoring mammography doses is underscored. Furthermore, it should be stressed that vendors using different methods of estimating the organ dose make reporting the dose across systems unreliable, as the dose reported by the three known methods differ by up to 19% [20]. Nonetheless, with vendors using various algorithms, some of which are not particularly well defined, there is a need for further work to establish a benchmark and allow for the comparison of doses between systems. Prioritizing manually calculated MGDs over system-reported ODs in mammography protocols can significantly influence patient safety and imaging quality [21]. By focusing on MGDs, which target glandular tissue, healthcare providers can better assess radiation risk and optimize dose settings. This shift could lead to more personalized screening protocols, especially for populations with higher radiosensitivity, ultimately enhancing patient safety.

The selection of an optimal dosage metric is paramount, influencing both patient safety and imaging quality [22,23,24] techniques. The high correlation observed between system-reported ODs and manually calculated MGDs overall, as well as across various centers and manufacturers, provide evidence that the MGD is a valid and reliable method to estimate radiation doses comparable to the conventional method using ODs. However, since the MGD considers various factors that influence the radiation dose and ensures a more comprehensive assessment of the dose absorbed by the breast tissue [25], MGDs can be concluded as a better measurement technique compared to system-reported ODs, especially in settings where multiple manufacturers are utilized.

To effectively integrate MGDs into clinical practice, we recommend developing standardized protocols that prioritize validating and unifying the MGD calculation method. Radiology departments should implement comprehensive training programs for radiographers, focusing on techniques that optimize MGDs. This approach ensures that efforts to minimize radiation exposure do not compromise the effectiveness of mammography as a screening tool.

## 5. Limitation

Although our study provides valuable insights into mammography dosimetry in Dubai’s healthcare settings, it is important to acknowledge several limitations that may affect the generalizability and interpretation of our findings. These limitations offer opportunities for future research and improvements in mammography practice. Firstly, this study focuses on Siemens and GE technology, reflecting their prevalent use in Dubai, which may limit the generalizability of our findings. Future research should include a broader range of manufacturers to better understand dose variability across different technologies. Additionally, the reliance on Ki values from the dose survey system introduces uncertainty in MGD calculations. Validating these values against direct physical measurements in future studies would enhance accuracy. Variability in equipment calibration and radiographers’ skills across clinics can also affect dose consistency. Standardizing calibration protocols and enhancing radiographers’ training are recommended to reduce variability.

## 6. Conclusions

Our study highlights the critical importance of accurate dosimetry in mammography, mainly in multi-vendor settings. The significant discrepancies understood between manually calculated mean glandular doses (MGDs) and system-reported organ doses (ODs) highlight the need for standardized dose assessment methods across manufacturers. Notably, this research does not definitively establish which dose metric is essentially more accurate, as both have strengths and limitations depending on specific clinical contexts. The observed variability in dose measurements across manufacturers and equipment age highlights the need for a standardized approach to mammography dosimetry. Our findings highlight the importance of regular quality assurance protocols and the potential benefits of transitioning to MGD-based dosimetry, particularly in multi-vendor settings. This shift could lead to more accurate dose monitoring and enhance patient safety. Moving forward, we recommend implementing equipment-specific quality assurance processes and involving qualified medical physicists in equipment procurement and upgrade decisions. These steps will help ensure consistent and accurate dose reporting across all machines, regardless of the machine’s age and other specifications, eventually improving the reliability of mammography screening programs.

Future research should focus on validating these findings across a broader range of manufacturers and developing standardized MGD calculation protocols that account for variations in breast composition and thickness across diverse populations. This approach will lead to more accurate dose monitoring, enhance patient safety, and improve the efficacy of breast cancer screening programs. Importantly, any dose reduction efforts must not compromise diagnostic image quality. By addressing these challenges and implementing these recommendations, we can significantly improve the accuracy and consistency of mammography dosimetry, ultimately enhancing patient care and safety in breast cancer screening programs.

## Figures and Tables

**Table 1:** Specifications of mammography machines used in the study.

Modality Location	Fabrication Year	Installation Date	Manufacturer	Model	Software Version
Screening Center 1	2011	20 September 2012	Siemens Medical	Mammomat inspiration	VB30
Screening Center 2	2011	20 September 2012	Siemens Medical	Mammomat inspiration	VB30
Screening Center 3	2011	20 September 2012	Siemens Medical	Mammomat inspiration	VB30
Screening Center 4	2015	2 January 2016	Siemens Medical	Mammomat inspiration	VB60
Screening Center 5	2015	21 March 2016	Siemens Medical	Mammomat inspiration	VB60
Diagnostic Center	2015	26 January 2016	GE Healthcare	Seno Essential	ADS_53.40

**Table 2:** Calculation methods and granularities known to be used by each system included in five examples of different manufacturers.

	Displayed Organ Dose	
Manufacturer	Calculation Method	Granularity
Philips (Eindhoven, The Netherlands)	Dance	Unknown
GE (Buc, France)	Wu	Proprietary measure
Hologic (Marlborough, MA, USA)	Boone	Unknown
Fujifilm (Tokyo, Japan)	Dance	Unknown
Siemens (Erlangen, Germany)	Dance	Unknown

**Table 3:** Patient demographics and analysis of study variables.

**Characteristics**			**Value**
Number of patients			2754
Age (years, mean ± SD)			51.74 ± 7.30
Breast thickness MLO view (mm, mean ± SD)			62.26 ± 11.96
Breast thickness CC view (mm, mean ± SD)			57.36 ± 10.70
**Variable**	**MLO View (Mean ± SD)**	**CC View (Mean ± SD)**	***p*** **-Value**
Organ dose (mGy)	1.38 ± 0.45	1.22 ± 0.38	<0.001
Mean glandular dose (mGy)	0.96 ± 0.37	0.81 ± 0.33	<0.001
Correlation between OD and MGD (r)	0.8	0.792	<0.001

MLO = mediolateral oblique; CC = craniocaudal; OD = organ dose; MGD = mean glandular dose.

**Table 4:** Patient demographics and analysis of study variables.

Variables	Mediolateral Oblique (MLO) View						Craniocaudal (CC) View					
	System-Reported Organ Dose	One-Way ANOVA (*p*-Value)	Calculated Mean Glandular Dose (MGD)	One-Way ANOVA (*p*-Value)	Pearson Correlation	*p*-Value	System-Reported Organ Dose	One-Way ANOVA (*p*-Value)	Calculated Mean Glandular Dose (MGD)	One-Way ANOVA (*p*-Value)	Pearson Correlation	*p*-Value
Overall	1.38 ± 0.45		0.96 ± 0.37		0.800	<0.001	1.22 ± 0.38			0.81 ± 0.33	0.792	<0.001
centers												
Screening Center 1	1.29 ± 0.44	44.06 (<0.001)	0.87 ± 0.28	108.19 (<0.001)	0.976	<0.001	1.16 ± 0.36	63.99 (<0.001)	0.75 ± 0.24	11.06 (<0.001)	0.920	<0.001
Screening Center 2	1.54 ± 0.50		1.06 ± 0.33		0.970	<0.001	1.32 ± 0.38		0.84 ± 0.27		0.874	<0.001
Screening Center 3	1.14 ± 0.36		0.77 ± 0.23		0.968	<0.001	1.02 ± 0.31		0.65 ± 0.19		0.908	<0.001
Screening Center 4	1.26 ± 0.38		0.84 ± 0.29		0.876	<0.001	1.09 ± 0.31		0.68 ± 0.22		0.902	<0.001
Screening Center 5	1.49 ± 0.46		1.01 ± 0.37		0.842	<0.001	1.41 ± 0.44		0.96 ± 0.37		0.850	<0.001
Diagnostic Center 1	1.41 ± 0.24		1.49 ± 0.77		0.638	<0.001	1.28 ± 0.19		1.22 ± 0.62		0.612	<0.001
		*t*-test (*p*-value)		*t*-test (*p*-value)								
Manufacturer												
Siemens	1.38 ± 0.46	0.96 (0.327)	0.93 ± 0.33	328.01 (<0.001)	0.923	<0.001	1.22 ± 0.38	4.03 (0.045)	0.78 ± 0.29	272.26 (<0.001)	0.891	<0.001
GE Medical Systems	1.41 ± 0.24		1.49 ± 0.77		0.638	<0.001	1.28 ± 0.19		1.22 ± 0.62		0.612	<0.001

## Data Availability

The data presented in this study are available upon request from the corresponding author. The data are not publicly available for ethical purposes.
